# Experiences of service transitions in Australian early intervention psychosis services: a qualitative study with young people and their supporters

**DOI:** 10.1186/s12888-022-04413-0

**Published:** 2022-12-13

**Authors:** Alyssa Milton, Tacita Powell, Katrina Conn, Rochelle Einboden, Niels Buus, Nicholas Glozier

**Affiliations:** 1 Sydney School of Medicine (Central Clinical School), Faculty of Medicine and Health, University of Sydney, 94 Mallett Street, NSW 2050 Camperdown, Australia; 2 The University of Sydney and Australian Research Council (ARC) Centre of Excellence for Children and Families over the Life Course, Sydney, Australia; 3 Adolescent Court and Community Team Justice Health and Forensic Mental Health Network, Sydney, Australia; 4 NSW Department of Education, Sydney, Australia; 5 Faculty of Health Sciences, School of Nursing, University of Ottawa, Ottawa, Canada; 6 Susan Wakil School of Nursing and Midwifery, Faculty of Medicine and Health, The University of Sydney, Sydney, Australia; 7 School of Nursing and Midwifery, Monash University, Clayton, Australia; 8 Department Regional Health Research, University of Southern Denmark, Odense, Denmark

**Keywords:** Early psychosis services, EIP, Care transitions, Mental health, Service user involvement, Qualitative, Young people, Families, carers and support people, Australia

## Abstract

**Background:**

Different Early Intervention Psychosis Service (EIPS) models of care exist, but many rely upon community-based specialist clinical teams, often with other services providing psychosocial care. Time-limited EIPS care creates numerous service transitions that have potential to interrupt continuity of care. We explored with young people (YP) and their support people (SP) their experiences of these transitions, how they affected care and how they could be better managed.

**Methods:**

Using purposive sampling, we recruited twenty-seven YP, all of whom had been hospitalised at some stage, and twelve SP (parents and partners of YP) from state and federally funded EIPS in Australia with different models of care and integration into secondary mental health care. Audio-recorded interviews were conducted face-to-face or via phone. A diverse research team (including lived experience, clinician and academic researchers) used an inductive thematic analysis process. Two researchers undertook iterative coding using NVivo12 software, themes were developed and refined in ongoing team discussion.

**Results:**

The analysis identified four major service-related transitions in a YP’s journey with the EIPS that were described as reflecting critical moments of care, including: *transitioning into EIPS; within service changes; transitioning in and out of hospital whilst in EIPS care; and, EIPS discharge.* These service-related transition affected continuity of care, whilst within service changes, such as staff turnover, affected the consistency of care and could result in information asymmetry. At these transition points, continuity of care, ensuring service accessibility and flexibility, person centredness and undertake bio-psychosocial support and planning were recommended. State and federally funded services both had high levels of service satisfaction, however, there was evidence of higher staff turnover in federally funded services.

**Conclusion:**

Service transitions were identified as vulnerable times in YP and SP continuity of care. Although these were often well supported by the EIPS, participants provided illustrative examples for service improvement. These included enhancing continuity and consistency of care, through informed and supportive handovers when staff changes occur, and collaborative planning with other services and the YP, particularly during critical change periods such as hospitalisation.

**Supplementary Information:**

The online version contains supplementary material available at 10.1186/s12888-022-04413-0.

## Introduction

Early intervention psychosis services (EIPS) support and treat young people (YP; generally aged between 12 and 25 years, although service age limits may vary internationally) who have experienced a first episode of psychosis (FEP), and in some cases people deemed at Ultra High Risk (UHR) for developing psychosis. The goal of EIPS is to provide access to evidence-based interventions that support symptomatic and functional recovery as early as possible [1, 2]. The support EIPS typically provide include a combination of talking and medication therapies, psychoeducation, and psychosocial assistance including social, familial, employment, education and accommodation related support [1].

In Australia EIPS, where they exist, are provided through two systems existing in parallel [3]. In many areas, state/territory funded health services provide EIPS as part of community mental health care services, often by specific EIPS teams, linked to geographically bounded hospital services. For example, in New South Wales there are 19 EIPs teams at 17 sites [4]. Additionally, in 2014 a national (federal) government initiative established six ‘hub and spoke’ *headspace* centres to deliver EIPS using the Early Psychosis Prevention and Intervention Centre (EPPIC) Model [5]. A potential benefit of establishing these federally funded EIPS was that they can provide integrated early intervention treatment SP the same service provides treatment for both UHR and FEP groups. These programs, delivered by non-government lead agencies, sit outside of the state-based system and have experienced insecure short term funding cycles throughout program establishment.

Bespoke time-limited EIPS were designed to enhance service quality and satisfaction for YP. However, they can result in a greater number of service transitions between child and adult mental health services. Such transitions can pose challenges to maintaining continuity of care. In general health services, the transition from paediatric to adult health care services is recognised and addressed with specific transition teams [6] that encourage the YP to take increasing responsibility and engagement in their care as they transition. In mental health services, however, YP take many different pathways through care, and fragmented delivery systems create potential for YP to ‘fall through the cracks’ between these services [7]. The recent Australian Productivity Commission Report on mental health emphasised that people experiencing mental ill health and their families struggle to navigate Australia’s overly complex mental health system [8]. There is a distinct dearth of research examining YP’s and their support people’s (SP; such as carers, parents and partners) perspectives on their service transitions through federal and state funded EIPS.

Recommendations for best practice in EIPS emphasise the need to involve YP in the planning, implementation and evaluation of services [2, 9]. Such involvement should be extended to SP (e.g., family member, partner or carer) as they are an integral part of the care system [7, 10]. Evaluation-based qualitative research can support YP and SP to provide rich and critical feedback for service improvement based on their lived experience.

International qualitative research, chiefly from Australia, the United Kingdom and the United States, has highlighted transition to and from EIPS at service entry and service discharge can be a challenging time [11–18]. However, there remains a paucity of Australian qualitative research that explores how YP and their SP experience EIPS support across all stages of their journey through the service. Most Australian qualitative research in this area has focused on engagement [14–17] and experience of EIPS access [18]. This an important and growing area of research, with a meta-synthesis of qualitative research describing how engagement is experienced by young people and carers [14]. The synthesis suggests there are five key themes including the experiences of finding help (which was often shrouded in uncertainty, distress, stigma and difficulty finding the right service first time, but relief once the right service was found), the factors that promote engagement (such as outreach, opportunities for peer support, making sense of the experience, and service level factors such as a youth friendly environment, easy access and flexibility) the therapeutic relationship with the clinician, the role of caregivers in supporting engagement, and the factors impacting ongoing engagement (such as poor initial help-seeking experience, internalised stigma, ongoing symptoms, the desire to solve one’s own problems, and other competing factors such as job-seeking appointments taking priority). Although service transition points are touched on in this qualitative research on EIPS engagement (especially at initial service engagement), additional research is needed to look at the full breadth of service transition points and experiences of continuity of care. The current research addresses this research gap using a qualitative and an evaluation-based approach. Specifically, this study aimed to explore the experiences and recommendations of YP and their SP with regard to:

i) Accessing and receiving support from federal and state funded EIPS;
ii) How EIPS interface with other social and health services, including hospital, as they move through the EIPS care pathway.

## Method

### Design

The design was 1:1 semi-structured interviews that are analysed thematically [19]. The study applied a critical realist orientation [20]. This study draws on qualitative interviews that were conducted as part of the Early Psychosis Youth Services (EPYS) Evaluation project [3], an independent evaluation of the 6 *headspace* early intervention psychosis services, commissioned by the Federal Government with partners EY (Pty Ltd), the University of Sydney, and The George Institute for Global Health. Acknowledging its challenges [21], we made use of the consolidated criteria for reporting qualitative research checklist (COREQ checklist [22]). Please see Supplementary File 1.

A qualitative semi-structured interview schedule was developed by the research team, including mental health clinicians and researchers (including a researcher with a lived experience of accessing an EIPS in Australia as a client, and a SP) from a diverse range of demographic backgrounds (cultural, gender, region). The interview schedule was based on questions from the first (unpublished) phase of EPYS evaluation, but additional questions about hospitalisation experiences and functional outcomes were added by the research team for this research stage to obtain a broader picture of the EIPS experience.

### Setting

The study took place in six community-based early psychosis services located across Australia. They included services from three of the new federally funded multidisciplinary FEP/UHR *headspace* services (Western Sydney and Darwin) and three state funded services in Sydney, one of which, in Western Sydney, covered the same geographical area as the federally funded services. Two services were located in urban areas of inner Sydney which has a high student population and a high proportion of employed professionals. Three services were located in Western Sydney, which is an area with a high number of people born outside of Australia. One service was located in Darwin, a regional city with a large Aboriginal and Torres Strait Islander population. For context, the way a YP can access EIPS varies. In NSW, a YP is allocated to a state or federally funded EIPS service via an assessment process initiated through the NSW mental health access line. Allocation is determined by whether the YP in under a community treatment order, the risk profile of the YP, practitioner choices and services relationships. The choice is not made by the YP or family. A YP or family can initiate a referral themselves to a federally funded service, however, admission into the program is at the discretion of the service. In Darwin there is no state-based service. In NSW, when state and federal services operate alongside each other, state-based services generally accept clients presenting with more severe illness.

### Participants

Participants were YP and their SP accessing a participating EIPS. The eligibility criteria for young people included: 1) aged 12–25 years; 2) clinician nominated; 3) minimum two week service engagement; and, 4) provided written consent (noting that those aged between 12 and 15 years required additional written parent or guardian consent; and those aged between 16 and 18 years required additional written parent or guardian consent when advised by the clinician).

The eligibility criteria for family or carers included: 1) being 18 years of age or over; and, 2) being a parent, guardian, family member, partner or friend of a current EIPS client.

### Recruitment and consent

Eligible participants were recruited through clinician referral. This referral process was selected as it enabled greater reach to the full cohort of clients and supportive others than purely passive recruitment (such as solely advertising the study via posters), but also provided and additional layer to ensure that participants were explained the research by someone they already had an existing trusting relationship with (i.e., their clinician), gave them time to consider whether they would like to take part in the research, and ensured the researchers remained at arms length during recruitment so as to ensure they did not feel subject to coercion or pressure in deciding whether to participate. This process was in keeping with the National Statement on Ethical Conduct in Human Research 2007 (updated 2018).

To enable participation in the research, clinicians were asked to nominate and invite all clients (and their SPs) on their caseload who met criteria to participate. Researchers communicated to clinicians that there were also seeking representation from special interest groups through a purposive sampling approach. Specifically, purposive sampling [19] was used to recruit a diverse sample of the EIPS client population and their family or carers who had an experience with the hospital system either personally or via the young person they support. Special interest group of clients and SP represented various clinical stages (UHR, FEP), ages, genders, culturally and linguistically diverse and Aboriginal or Torres Strait Islander backgrounds. The researchers fed back to the coordinating clinicians at each EIPs if there were any recruitment gaps and clinicians checked their entire caseload for eligibility so as to minimise the potential for gatekeeping and bias.

A two-stage consent process was applied where clinicians briefly described the study to potential participants meeting eligibility criteria gaining consent to refer to the researcher. The clinician scheduled the interview for those expressing interest in participating. Prior to interview, participants had the opportunity to review the participant information and consent forms, and discuss any questions, before giving informed consent. Participants aged 12–18 years required parental/guardian co-consent, with 16–18 year olds parental/guardian consent being subject to clinician advice and state specific laws.

### Data collection

A priori sample size estimates was guided by research [23, 24] suggesting 20–40 participants would be required for data saturation as the research involved recruiting a non-homogenous participants (i.e., both YP and SP) and was run across multiple EIPS settings across Australia with different funding (state and federal).

After informed consent, audio-recorded interviews were conducted face-to-face at the EIPS or via telephone between Dec 2019 and May 2020. A SP could be present for the interview if requested by the participant. An interview guide (covering client experience of: coming into the program; the program delivery; the impact of the program on their functional outcomes; hospitalisation experience whilst involved in the program; treatment; ongoing community care; and auxiliary programs) is provided in Supplementary File 2 and 3. Interviews were conducted separately by two people (1. a psychologist and qualitative researcher; and 2. a psychiatry registrar (i.e., a psychiatry trainee)) both with experience working with EIPS but had not worked at any of the participating EIPS. The research team supported the interview phases through iterative feedback. The average interview duration was 57 minutes (Standard Deviation = 18 minutes) for YP and 68 minutes (Standard Deviation = 17 minutes) for SP. Participants were compensated for their travel expenses up to $25 AUD, noting participants with circumstances requiring to travel long distances and incur costs exceeding this amount being assessed on a case-by-case basis (although this case did not arise during the research period). Participants could choose the method of reimbursement, with all selecting a $25 AUD supermarket voucher.

### Analysis

Interviews were transcribed using the NVivo Transcription Service, then anonymised and checked for accuracy by four members of the research team. Interviews were transcribed as they were conducted and the team met weekly throughout the interview process to engage in iterative discussion about the main themes emerging, and sampling requirements. As it was part of a larger evaluation, we were not able to assess data saturation via a stopping criterion, however, we as described above relied on a priori estimates of an appropriate sample size to reach saturation. Data were interpreted thematically using an established six step process of qualitative analysis [25] by the research team. Thematic analysis provides a flexible method of analysing and interpreting substantial amounts of qualitative data. The analysis enables exploration of commonalities and variations across subsets of data—such as data provided by different stakeholder groups or collected in different settings. The six steps include: (1) *Become familiar with the data*: the team members were very familiar with the data as they had checked the transcription data for accuracy and engaged in weekly discussions about the themes, and also analysed the data separately in the initial EPYS evaluation (which used top-down framework [impact, satisfaction and culture, and system] to organise inductive findings. Noting that the research reported here was a separate purely bottom-up inductive analysis); (2) *Generate initial codes*: the research group initially explored a sub-sample of data by making comments in the participants own words in Microsoft word document of the de-identified transcripts to develop a preliminary coding framework; (3) *Search for themes*: Open coding was conducted using NVivo 12 Software by one of the analysis team members; 4 & 5) *Review and define themes*: the themes in the coding framework continued to be collaboratively refined and named through an iterative process of reading, coding, reflection and discussion in the weekly team meeting until all significant parts of the data had been considered and a codebook was collaboratively developed which included sub-themes and overarching themes. All interviews were subsequently double coded. The collaborative approach to analysis supported reflexivity as it encouraged comparisons and sharing of diverse perspectives the research group offered with their various backgrounds and lived experience [26]; and, 6) *Write-up*: the results were written up and reviewed by all team members. A lay-summary of findings were returned to participants, however, there was no formal opportunity for participants to feedback on the findings and recommendations other than contacting the researchers directly.

## Results

### Participant background

In total, 27 YP and 12 SP took part in the study. Only one YP decided not to participate after an initial clinician referral. The number of participants who declined at clinician invitation was not recorded. Two YP requested a support person to be present who also participated in the interviews. Nearly all (11 of 12) SP were supporting a YP who was also interviewed. Participant demographics are presented in Table 1.

**Table 1:** Participant Demographics

	Young people		Support people	
	*Median*	*Range*	*Median*	*Range*
Months in program	15	1 to 55	12	6 to 50
	*n*	*%*	*n*	*%*
EIPS service type				
State Funded	8	30	2	17
Federally Funded	19	70	10	83
EIPS locations				
Inner Sydney	7	26	2	17
Western Sydney	15	56	7	58
Darwin	5	19	3	25
Age				
14–16 years	3	11	-	-
17–19 years	6	21	-	-
20–22 years	9	32	-	-
23–25 years	9	32	-	-
26 years	1	4	-	-
Gender				
Male	15	56	4	33
Female	12	44	8	66
Other	0	0	0	0
Living situation				
Family	19	70	-	-
Partner/husband/wife	2	7	-	-
Friend(s)	2	7	-	-
State appointed carer	1	4	-	-
Own	1	4	-	-
Not specified	2	7	-	-
Aboriginal or Torres Strait Islander				
Yes	3	11	0	0
No	24	89	10	83
Not specified	0	0	2	17
Language other than English				
Yes	9	33	9	75
No	18	67	3	25
Education, training or employment				
Yes	13	48	7	58
No	6	22	3	25
Not specified	0	0	2	17
Currently in education				
Yes	17	63	-	-
No	10	37	-	-
Currently in employment				
Yes	10	37	-	-
No	17	63	-	-
Clinical status				
UHR	5	19	-	-
FEP	22	81	-	-
Relationship to client				
Parent or Guardian	-	-	11	92
Partner	-	-	1	8
Other family member or friend	-	-	0	0

### Transition points

**Fig. 1:**
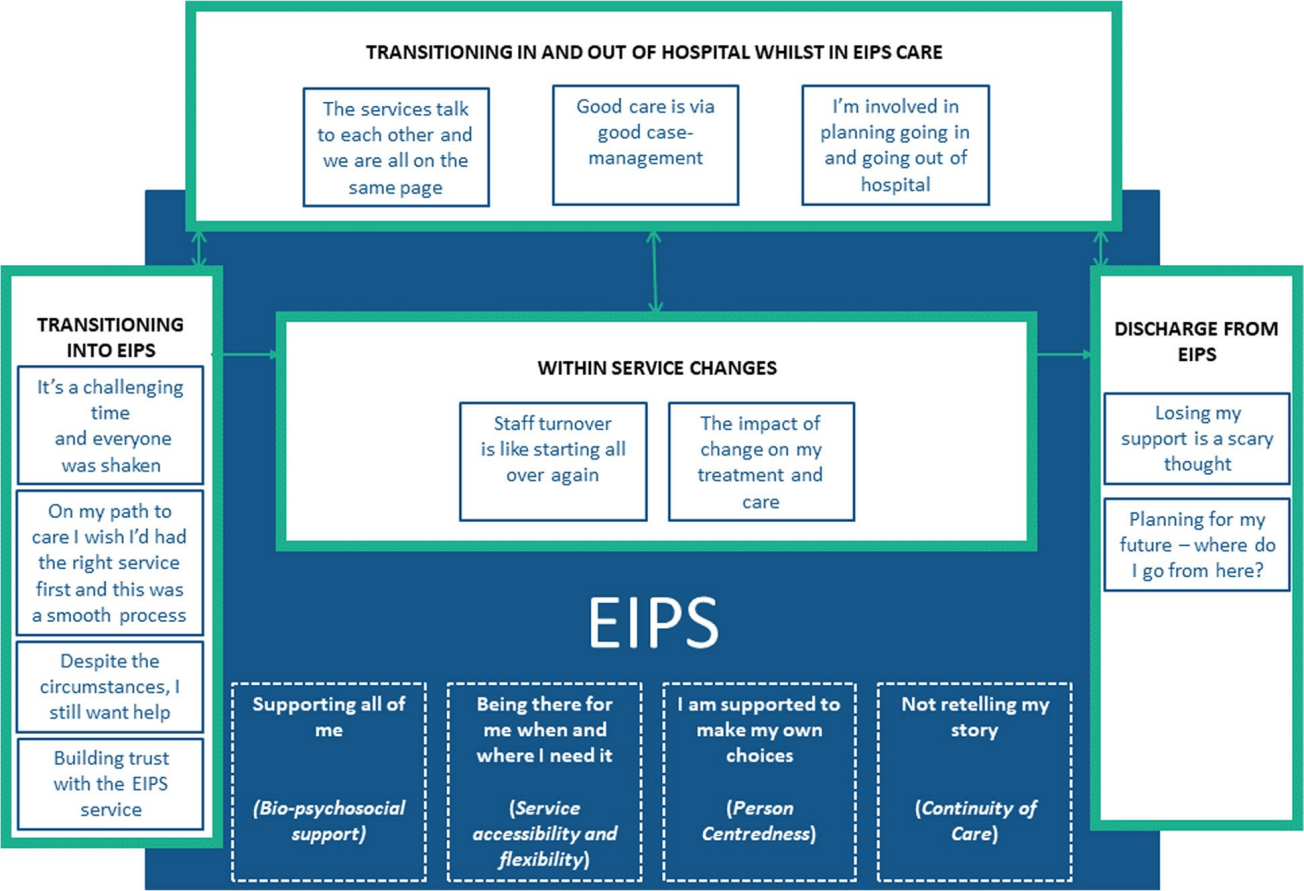
Service Transition themes and subthemes

As shown in Fig. 1, there were four major service related transitions in a young person’s journey with the EIPS identified that reflected critical moments of care and its continuity. These included *(1) transitioning into EIPS; (2) within service changes; (3) transitioning in and out of hospital whilst in EIPS care; and, (4) EIPS discharge.* Participants’ desire for EIPS program delivery to support the whole person not just their mental health and engage in planning around this (bio-psychosocial support), be there whenever and wherever they needed it (service accessibility and flexibility), place them at the centre of decision making and choice (person centredness) and be sufficiently coordinated so they did not have to continuously retell their story between and within services (continuity of care) were subthemes that underpinned all the transition themes.

The solid blue box represents the EIPS. Service transition themes are indicated as boxes with a green outline and white interior. Transition subthemes are indicated as boxes with a blue outline and a white interior. EIPS program delivery subthemes are situated within the EIPS blue box and are indicated by boxes with a white dashed outline.

### Transitioning into EIPS

Before engaging with the EIPS, nearly all participants (YP and SP) described their situation as an extremely challenging time due to symptoms of mental illness, changes in behaviour, decline in functioning and situational stressors. Further, half of all participants described delays in accessing treatment or seeking help. Prior to engagement with the EIPS, all YP and SP reported that they (or the YP they support) had accessed at least one other service for mental health support — chiefly provided by the hospital system (approximately half), followed by private psychologists, GPs, private psychiatrists and a small number accessing school counsellors.

About half the participants reported negative initial help seeking experiences with other services in the community (private psychologist and psychiatrists, GPs, and school counsellors) — typically attributed to the service not being youth friendly, not being useful, or focusing on the wrong symptoms or diagnosis.

*“…like I had only really seen a psychologist once before and that was like useless (…) Every session we’d move further and further away from the point.”****YP P29***

Approximately half of the YP and SP in this sample reported having a *hospital admission* prior to, and triggering the EIPS engagement. YP frequently described their situation as “*scary*” in the lead up to, and during, hospitalisation prior to receiving support from the EIPS, and SP described the situation as incredibly stressful. Subsequent transitions into the EIPS from hospital were described as a time of confusion and uncertainty by over half of the YP and SP.

The majority of these YP who had previously been hospitalised and their SP reported the referral process between hospital and the EIPS team was reasonably well integrated and generally met their needs. The vast majority were referred via hospitals that had established relationships with the EIPS. There were, however, a small number of occasions where the YP or SP reported that the process of referral between hospital and EIPS was disjointed and/or delayed.

*“I remember actually getting quite frustrated because I was meant to get discharged a couple of days earlier, but I couldn’t get discharged until the doctor got in contact with [EIPS] and I was just delayed*. *I just wasn’t sure whose fault it was. But I just felt it was a bit disorganized.”****YP P11***

Despite their initial experiences (positive, negative or mixed) with other community based or hospital services, the vast majority of all participants recognised that they wanted some form of help at the time of EIPS engagement. A small number of YP described having some level of EIPS service engagement hesitancy, often because of their mental state, the feeling the assessment process was intrusive, or because of shame or stigma. The role of their SP (usually a parent) to assist with help seeking in this early stage was emphasised by some of the YP who expressed engagement hesitancy.

*“I remember sort of at the start I wasn’t too happy about coming into [EIPS], probably because at the first interview, like I think it’s more related to my insecurities, though I probably divulged a bit too much information that just made me go like, oh, why are they asking me or these questions about these random things.”****YP P9***

*“I wasn’t super willing to go [to the EIPS] at the beginning. It was more of my mum wanted me to go. But then, I guess, slowly I began to, I kinda was like, ‘yeah, okay, this is okay’, and I guess I wasn’t expecting that much out of it.”****YP P31***

Once engaged, almost half of the YP and SP described not knowing what to expect from the EIPS. They did not know what mental health support would look like, felt like they were lacking necessary knowledge and expressed a need to understand their situation and symptoms.

*“I don’t think anyone else from my family has like, seen like a counsellor or a psychologist or anything. I really didn’t know what to expect. I just, I don’t know, I knew that I was upset, and I don’t know I wasn’t feeling any better then. I don’t know, I guess I wanted help.”****YP P7***

After their first meeting with the EIPS, YP and SP predominantly reported positive experiences. *Establishing the relationship* was seen as critical, particularly due to the YP feelings of vulnerability prior to engagement. YP often described the positive impact they experienced from building this rapport, which including a sense of relief being able to opening up and share their story.

*“I found it very calming. Knowing I could share my whole story. Like, consider the whole information about myself by piece by piece. Chapter by chapter.”****YP P12***

Reflecting on these initial experiences, engaging with YP, giving them an overview of the service and building the relationship could be done through outreach and in creative ways that met them where they were at.

*“[At the hospital] I felt very comfortable with [EIPS staff] around. We played some cards (…) She came in with cards (..) So I was so happy to see [EIPS care coordinator]. She was telling me about [the EIPS] and that it could help me.”****YP P21***

However, about a third of YP and SP highlighted that it took time to adjust to the EIPS support provided and also adjust their own expectations.

*“At first I was very hesitant to trust anyone, to be honest. By yeah after some time I warmed up to the people I am talking to now.”****YP P35***

*“I’m a mother and I was really desperate. And I was fearing for his life. I thought I thought they would, they would you know, they would give me a roadmap. And I thought it would be sort of, you know, like, easier to diagnose and everything. And, of course, you know, like they had to take it slowly.”****SP P30***

### Within service changes

Staff turnover of the care-coordinator and treating psychiatrist was discussed by almost half of all participants. The frequency of staff turnover varied for each individual, with the most extreme circumstance being “*more than ten*” (SP P22) care-coordinators over a three year period. High levels of staff turnover were more frequently reported by those attending federally funded EIPS, and there was much greater stability, especially of senior clinicians (such as consultant psychiatrists) in the state funded services. Turnover was attributed by SP and a small number of YP to staff burnout, workplace culture, insecure funding of federally funded services, and the structure of the health system—such as regular psychiatry registrar rotation (although 6 month rotation is the same across state and federal services).

*“And I don’t know if that’s because it’s government funded or of the management. I don’t know. So I feel like that you’re always kind of on edge because I’m always thinking, “what if [case manager] leaves?” because that rapport takes a long time. So I think if that is due to the management, I think that maybe their culture could change.”****SP P3***

The impact of staff turnover on YP and SP was predominantly negative, with participants describing feeling angry, uncomfortable, sad, and that the process was generally unsatisfactory as they felt “*passed around*” (YP 5). There were only a small number of circumstances where they felt they were given sufficient warning that the change would occur (e.g., well planned psychiatry rotations). A few participants described having come to terms with constant change being a normal part of the health system.

*“So I’m not really sure, psychiatrists here, especially in recent years, tend to sort of come and go and I think, doesn’t really have to do with them? I just think it has to do with like just the way that system is now.”****YP P20***

Staff turnover also created a noticeable breakdown in the young person’s consistency of care and therapeutic alliance. Participant’s spoke of “*falling through the cracks*” (YP P5) and losing ground after taking time to build trust and rapport. This left participants feeling like they had “*to start all over again with someone else*“ (YP P7) and retell their story. Conversely, those that had not experienced turn-over of their case manager often spoke very highly of the close relationship, for example describing themselves as “*lucky*” (SP P4; YP P12; YP P29). One participant described their case-manger as their “*ride or die*” (YP P21), reflecting how essential their support was.

Turnover had adverse impacts on some YP’s treatment consistency — as the staff, particularly psychiatrists, had different treatment and care approaches. For example, one young person described that psychiatrist rotation often resulted in unwanted medication changes.

*“Because you have to repeat the story and like and like by doing that, the new psychiatrist doesn’t really have a good idea of who you are. So like sometimes they change, and want to change your medication, but they don’t know you.”****YP P17***

Ensuring YP and their SP were provided with information about the staff handover before it took place, being on board with the upcoming change, and receiving some consistency in the support from other members of the team (or collective case management) eased the impact of the staffing change.

*“So the psychiatrist changed, so doctor [Dr 1 Name] left, but I started meeting up with doctor [Dr 2 Name], but, yeah, so I was informed the whole way, so I was happy, so it was not a surprise or anything. [Interviewer: So did it, how did it impact you?] It was sad that [Dr 1 Name] was leaving, because I hate change, so I wasn’t a massive fan of her going, but then eventually, I sort of got used to doctor [Dr 2 Name], you know, he’s very kind, so, but I still have [case manager] so yeah.”****YP P32***

### Transitioning in and out of hospital whilst in EIPS care

Participants’ experiences of the integration—that is, how embedded the EIS was in the hospital system which could promote better care coordination and communication—between the hospital and the EIPS were dependent on multiple contextual factors (Supplementary File 4). This included geographical (e.g., the geographical proximity of the hospital to the EIPS—noting EIPS that were closer to the hospital generally resulted in better experiences for young people due to higher level integration), temporal (e.g., what time of day the hospitalisation occurred—with daytime hospitalisation generally being associated with more coordinated hospitalisation experiences; the length of hospitalisation—with shorter hospital admissions sometimes getting missed by the EIPS), organisational (e.g., the culture of the hospital; the strength of the formal established link between hospital and the EIPS—noting state funded services formally shared staff between the EIPS and the hospital and used the same systems) and individual (e.g., the case-manager’s level of assertive engagement—with proactive case managers leading to experiences that felt more supported). How well the EIPS was integrated into the hospital system influenced the YP and their SP experience of transitions between the services. State funded EIPS generally were seen as having strong links with the hospital system, whereas the federally funded services received mixed reports.

Transitioning into hospital with support from an EIPS was ideally done in collaboratively — with the YP and their SP involved in the decision-making. This occurred was when a young person required closer monitoring in an in-patient hospital setting, such as when changing medication or when acutely unwell (noting some YP were able to be supported at home by the EIPS and avoid hospital admission completely “*I’m at home with people that I know, not at a hospital where nurses give you medication and what not, to calm you down and being around other unwell people and that. So, I guess it was better at home.”* ***YP 16***). In a joint interview, a YP and SP described:

*“YP P18: I don’t even remember ever feeling like “oh, no one is listening to me” because they all listen to me and my opinion and what I was feeling, you know, I always heard, felt like I was heard (…)*

*SP P19: I guess you sort of understand that when you’re going through that part of the hospital, there’s a point where I guess they [EIPS] just have to hand over, they can’t be that involved because the hospital’s got their own people in it. So they go ‘okay we will step back, but we’ll be ready. As soon as you’re ready, we’re ready’. And that’s what I felt like. They were ready to step back in at the exact right time.”*

EIPS coordination and communication with the hospital staff was vital. The strength of the relationships between the EIPS and hospital was frequently reflected in participants’ experiences. Specifically, participants reported better experiences when they were admitted to hospitals that had formal arrangements or were integrated with the EIPS — often this coincided with the EIPS’s physical proximity to the hospital.

*“For me, because my son, the first day we go in to, we go into the, admit by the hospital, [EIPS] people is already there. It was there. For my daughter’s case [admitted to a hospital out of area] they were not there”****SP P1***

When the EIPS was not involved in the hospital admission (usually out of hours), it was more challenging for the young person and their SP. In the small number of cases where a lack of communication and continuity of care at admission between the EIPS and the hospital was evident, it was reported as negatively affecting YP when they were at their most vulnerable.

*“I felt like it was needed that [the EIPS] was supposed to tell the hospital what was the scenario. But because [the EIPS] didn’t tell the psychologist, the inpatient psychologist about what happened. I had to explain the whole situation again. Which was really tiresome.”****YP P11***

Once in hospital, EIPS contact (either face-to-face visits or via phone) was appreciated and important to YP and SP. Such contact could assist YP and their SP with advocacy related concerns, promote greater continuity of care, decision making and choice, family involvement, and planning (mental health, relapse prevention, medication) and importantly it assisted the transition out of hospital. This transition out of hospital was aided by the strength of the relationship between the hospital and the EIPS.

*“So, they [hospital and EIPS] had constant communication, so I think it was very beneficial to both parties to be able to, you know, head back and forth like, you know, what they think that [young person] should be doing, and whether or not she should be leaving [hospital], and you know, I think it gave both parties confidence as well.”****SP P37***

The vast majority of YP and SP cited that the transition points in and out of hospital, and intensified follow-along support from the EIPS post hospitalisation were critical, but approximately half suggested that such transitions would benefit from a more consistent approach being applied. The experience of hospitalisation was highly dependent on the EIPS case manager supporting the YP — as their support style, coordination of care, knowledge of the client, communication and assertive engagement could vary.

*“I feel like it depends on your case manager. I actually don’t think that my first case manager when I went to my first hospital admission focused on it well (…) But my second and third admission, I had the same case manager. So it was it was much easier for me to do the mental health plan. The techniques were thoroughly taught. So it really depends on the case manager. Yeah, but the whole time I had the same psychiatrist. So she was really, well, great.”****YP P11***

Experiences of coordinated discharge planning that included the young person in the process varied greatly between participants—with some participants unaware of discharge plans, whereas others described a relapse prevention plan, schedule for follow-up, coordination with their family and external groups such as school, university, or housing support. Further, about one quarter of the participating YP emphasised the importance of the EIPS’s collaborative and informed review of their medication post hospital discharge, as they felt over medicated or experienced side effects from new hospital initiated medications.

*“My lithium dose was lowered [post hospital discharge] and then olanzapine made me feel really drowsy the entire time. So then I talked to my psychiatrist and she changed it (…) I talked to my parents and they said, oh, you should just tell the doctor that you’re experiencing any side effects, you change the medication. And I think the doctor gave us one or two or more options and talk about benefits, disadvantages and side effects.”****YP P11***

### Discharge from EIPS

While none of the participants in state funded EIPS were reaching their age limit or their maximum length of time they could spend with the EIPS (average time with state EIPS was 11 months), approximately a quarter of participants in the federally funded services, were at the cusp of service discharge (reaching the upper age limit or their maximum length of time with the program; average time with federal EIPS was 24 months). Discharge was identified as an area of concern for these YP and SP. Approximately a quarter of the participating YP and SP were reaching the end of their care with the EIPS. Half of these expressed concern about availability of support post discharge.

*“when this program finishes she has to go somewhere else, because I believe that there’s not going to umm, it is a long term thing so there will be some sort of ongoing support required. Yeah. That is the worry I have, once she finishes [with the EIPS], what do we do.”* ***SP P15***.

These YP and SP had not been engaged in planning their discharge whether this be to primary care or adult mental health services, despite knowing that they could be discharged soon.

*“I’m just scared for the future because I’m 25 now, so not going to be with [the EIPS]. So I haven’t actually talked to anyone about what I’m going to do.”* ***YP P17***.

A consistent approach to engaging YP and their SP in early and comprehensive conversations around planning and continuity of care when transitioning from EIPS to adult mental health services would be highly beneficial and provide *“peace of mind*” (SP P15). Further, most YP and SP who spoke of discharge or “*graduation*” (SP P4, YP P11) from the EIPS highlighted how it would be difficult to replace the EIPS, expressing a sense of loss, and stressing the importance of building good relationships with a new service.

*I will miss them [the EIPS] (…) I will lose, you know, the buffer I have got. I was just like, you know, if something happens and as she has a lot of thoughts then she will need somebody to talk to. Then I may have to find other sources. Maybe you may have to engage a psychologist. But, you know, it’s not that easy to find somebody, you know, to listen to you, she doesn’t know. Depends, you know, that is another type of relationship that you have to get along, to know each other before, you know it works, but that is why I am thinking positively, you know, if she graduates from [the EIPS], and then I’ll have a think about whether I can get other professional support.* ***SP P4***.

## Discussion

This is the first known qualitative study that concurrently explores YP’s and their SP’s experiences of engaging with EIPS in Australia. Service-related transition points (*transitioning into EIPS; within service changes; transitioning in and out of hospital whilst in EIPS care; and EIPS discharge*) were often critical junctures in a YP’s care pathway. How these service-related transitions were managed clearly affected the continuity of care of YP and their SPs. Within service changes such as staff turnover affected the consistency of care a YP and their SP experienced, which could result in information asymmetry, result in dissatisfaction and reduced trust, adversely impact treatment and care, and required time to rebuild. When YP and their SP experienced smooth transitions it was often due to EIPS staff taking steps to ensure continuity of care, service accessibility and flexibility, person centredness and provide bio-psychosocial support and planning. These overarching findings are in keeping with much of the Australian and international literature on service engagement [14].

Our findings highlight the challenging circumstances faced by YP when entering the mental health system for the first time — including experiencing problematic symptoms, a decline in functioning, situational stressors, and delays in accessing appropriate treatment. The literature highlights that one of the key ways that EIPS can improve outcomes for YP is by reducing the duration of untreated psychosis they experience before receiving treatment [27, 28]. However, in this research it was common for YP and SP to report poor first engagement experiences with other primary, secondary and tertiary health and mental health providers before reaching the EIPS. By the time participants in our study reached EIPS, they were often confused, in considerable distress or at crisis point, and many had been hospitalised, which is in line with other qualitative Australian findings on service engagement [14–16]. This is important as qualitative research has highlighted that a YP’s uncertainty about first episode psychosis is compounded when the first contact with a health provider is negative [27]. Although EIPS in Australia promote a ‘no wrong door’ policy [29], this clearly needs to extend further to ensure other primary, secondary and tertiary care services have greater awareness of both psychosis and the referral pathway to streamline access to EIPS. Once engaged, YP and SP reported better experiences, especially when EIPS are flexible and tailored to the young person’s needs in terms of when, where and how services were delivered.

Despite negative experiences prior to EIPS entry, YP in this study highlighted that building trust and a relationship was critical – and although it could take time, the EIPS was generally able to do this well. Person-centred support and communication, which was available, flexible, creative and involved YP in decision-making was highly recommended from the outset of engagement. This type of support is in line with other EIP-focused qualitative research findings that positive connections with a service, built on trust, emotional and informational support, open communication and shared decision making, led to better treatment engagement [14, 30, 31].

Occasions in this study where this person-centred care broke down primarily occurred when changes service changes arose. It is possible that service led communication and collaboration to keep the young person actively involved and centred on their needs can be challenging at these critical junctures— as services are scrambling to deal with service related factors such as staffing shortages or a crisis. However, EIPS need to be aware of the importance of addressing these key tension points— with appropriate planning and strategies that promote choice for the young person established as early as possible. Indeed, local and internationally policy emphasise that promoting choice a defining feature of a high quality service [32]. Other research indicates that greater treatment-related empowerment is an essential part of the recovery experience [33]. Ultimately, this not only includes YP (and where possible and appropriate SP) involvement in treatment related decisions, having clear and consistent treatment plans, having access to familiar clinicians potentially via a collective case-management approach—but also includes client involvement in planning when unforeseen changes occur.

In line with descriptions of EIPS in the literature [1], participants spoke of their EIPS providing medication-based therapies, counselling, psychoeducation, and psychosocial assistance including social, familial, employment, educational and other types of psychosocial support which is discussed in depth in our linked paper. Although treatment and bio-psychosocial planning are key fidelity items in the EIPS model of care [5], routine updating of plans was not evident for all participants at these key service transition points. EIPS need to ensure that consistent collaborative comprehensive treatment and bio-psychosocial assessment and planning including a focus on self-management with YP and their SP takes places routinely, especially when changes which might place tension on continuity of care—such as a hospitalisation—occur. Indeed when transitioning in and out of hospital. maintaining contact with the YP and SP (face-to-face or telephone), advocating for the YP, and facilitating the planning (advanced directives, admission planning, discharge planning, relapse prevention, treatment and bio-psychosocial planning) were essential ingredients of what was considered gold-standard support provision and continuity of care.

Continuity of care was critical to YP and SP in this study. In line with other health focused research [34], this continuity included relational (i.e., the EIPS–young person relationship), informational (i.e., the availability of information that is appropriate and enabling best practice and coherent care) and management (i.e., the consistency of care delivery).

Continuity of care was needed when transitioning between services (such as during a hospitalisation, and at EIPS discharge), and when staff in the EIPS changed. In relation to hospitalisation, understanding how YP experience the interface between hospital and an EIPS is a critical yet under researched area. In adult mental health settings, research has highlighted that care transitions involve a multitude of health and social care professionals working within and across different organizational boundaries [35]. With the movement of clients from intake to discharge from inpatient mental health settings being particularly highly complex [36]. This complexity was reflected in our findings where contextual factors (including geographical, temporal, organisational and individual factors) influenced the young person’s and their SP’s experience of hospitalisation. Furthermore, it is clear from these findings that the EIPS is helping the YP and their SP to navigate the system, and enabling smoother transitions at hospital admission and discharge by driving communication between the services, the young person and their SP—with above-mentioned planning process being an essential facilitator of these smooth transitions. Intensifying support provision, at hospital transition points is also recommended.

Participants in this study reported frequent changes in staffing. Staffing changes are a reality of mental health service provision. However, more can be done to actively address this at a management level with collaborative planning with YP and their SP when staff changes occur, and collective case-management to ensure at least some staff remain familiar when a staff member leaves. Staff working conditions may also need addressing, as participants highlighted that insecure funding models and burnout could increase staff turnover damaging care consistency yet further.

Some YP and their SP in federally funded EIPS were also concerned that the process of transition into adult mental health services was not sufficiently planned or communicated when approaching discharge. International research has highlighted this transition point between services is problematic [11–13]. For example, during this care transition YP report finding themselves ‘lost’ [37], they do not feel adequately prepared or supported, lack an understanding of how adult services function, feel a sense of loss, and experience acute worry over starting with an unfamiliar service [35]. Further, both YP and their SP report that their voices are not always adequately listened to during the transition process [35]. Importantly, poor transitions can significantly increase the likelihood of poor mental health outcomes into adulthood [37].

Bridging the gap between EIPS and adult mental health services is a priority. This current research shows that more could be done to ensure this transition into adult services in Australian settings is seamless— with YP and their SP more actively engaged in early planning. Like in general paediatric health services, services such as the Trapeze Model [6] that are teams specifically dedicated to supporting these service transitions may be beneficial in mental health. In addition, it is possible that EIPS may be able to increase the level of satisfaction and address the unmet needs of YP and their SP by having some flexibility in length of EIPS and service-age limits [38].

### Key recommendations

The perspectives and experiences of YP and carers can provide valuable insights for services improvement. This is especially important as this process moves beyond tokenism to a place where real action can be taken [39]. Key recommendations based on the lived experiences described in this study sample, which relate to service transitions, are provided in Table 2 and highlight the important actions services can take to improve EIPS support. This list was collated by the research team, which includes lived experience perspectives, but these recommendations could be further enhanced through ongoing participatory design processes with YP and their supporters to enhance service delivery.

**Table 2:** Key recommendations across EIPS transition points

Transition point	Key Recommendations
**Transitioning in to the EIPS**	• Acknowledge that this is a challenging time for the young person and their families, which may be because of psychiatric symptoms and functional issues, negative experiences with their previous engagement with the mental health system, stigma, and that the process may feel intrusive. • Establish strong pathways into the EIPS to ensure transitions from other services are transparent, coordinated and communicated well. • Where possible include support people in the process. • Provide a clear overview of the EIPS support and treatment. • Seek to understand and address the young person and their supporters’ expectations of care and support needs. • Work towards building trust, rapport and a supportive relationship. • Use creative engagement strategies that support young people to feel at ease. • Provide iterative and ongoing communication about the process and what is likely to happen with treatment and support, acknowledging the common challenges and benefits from the outset and that it takes time to adjust.
**Transitioning in and out of hospital whilst in EIPS care**	• The EIPS involves young people (and where appropriate their support people) in decisions about hospital transitions (entry and discharge). • EIPS coordination and communication with the hospital system is active and ongoing to maintain a young person’s continuity of care. • Collaborative discharge planning occurs routinely and includes relapse prevention planning, scheduling follow-up EIPS support, and coordination with other involved parties (support people, and external groups such as school, university, or housing support). • The EIPS routinely review the prescribed medication after the hospital discharge to assess changes in dosage and check for side effects from any new medications.
**Within service changes**	• At EIPS commencement, explain to young people and their supporters how the staffing system works (e.g., regular 6 month psychiatry registrar rotations) and the handover process when staff changes occur. • Minimize staff turnover where possible. This might be done through promotion of better workplace culture, initiatives to reduce staff burnout, workplace funding stability. • Ensure processes to reduce the impact of staff changes are in place—such as team-based approaches to care coordination, collective case-management, regular team case reviews to ensure team collaboration, and supervision over individual cases. • If staff turnover must occur, ensure that the young person and their supporters are aware of the change as early as possible, and are included in the decision making prior to the event arising. Best practice recommendations at this time include sufficient provision of information and support, joint handover meetings and maintaining consistency of other team members. • If a new psychiatrist or registrar commences, they should ensure that any changes to the young person’s medication does not happen abruptly without full consultation and joint decision making taking place.
**Service discharge**	• The length and scope of service, and pathways to other mental health services in the future, are outlined comprehensively when the young person commences with the program. • Young people and their support people are included in the discharge planning as early as possible when a young person is approaching EIPS discharge. • Discharge should be well-planned and coordinated, with communication between EIPS and adult mental health services ensuring that the young person and their support people have optimal continuity of care.

### Strengths and limitations

A strength of this study is that it included a relatively large sample size across multiple service locations and used purposive sampling to maximise the representativeness of participant backgrounds. We had good representation across age groups, from people who identified as having an Aboriginal or Torres Strait Islander background, from people who spoke a language other than English at home, and from people engaged (or not engaged) in Education, Training or Employment (See Table 1). However, we only had representation from people who identified as males and female and did not have representation from people who identified with another gender group.

Other qualitative Australian EIPS research has been limited as it did not recruit YP who struggle to engage with the service or had fully disengaged [16]. Similarly, although in this current study the researchers requested this type of contact be made, it could not be guaranteed as participants were recruited through EIPS clinician nomination as part of a larger evaluation. Therefore, it is possible that fewer individuals with negative service experiences or those that had disengaged with the EIPS were recruited. For those that were recruited to participate, the researchers conducting the interviews were not associated with the involved EIPS, thus the participants’ willingness to reflect openly on the service may have been enhanced. The current research findings are also limited to the views of YP and SP who were approaching discharge only. Further research is needed to gain a more comprehensive picture of the EIPS discharge process as we only recruited YP and SP who were still receiving EIPS support.

There are also general limitations to this research. Firstly, like most qualitative studies, including those focused on EIPS [40], the interviews rely on participant recollection, where challenges with recall may impact findings. Secondly, as this is Australian research, the findings— particularly relating to federal and state funded services— may be limited to the Australian context.

A final limitation to this research was that participants were provided a lay summary of findings and recommendations, however, there was not a formal opportunity for participants to directly feedback outside of contacting the researchers directly. Our analytic team did, however, included a lived experience researcher and a SP, this may help to triangulate findings and provide important insights from the perspective of those who have used EIPS and other mental health services.

## Conclusion

A key priority in many countries— including Australia— is to improve child and youth mental health systems because they risk falling through the cracks in fragmented delivery systems [7]. Clearly, there remains work to do, with greater attention in policy and practice needing to be paid to these key transition points YP experience. This research extends the current qualitative research [14] on service engagement and offers some insight into how YP and their SP experience service transitions, providing potential strategies the EIPS can implement to improve the pathway from service entry to service discharge and beyond.

## Supplementary Information

**Additional file 1:** **SupplementaryFile 1.** COREQ checklist. **SupplementaryFile 2.** Interview Guide for Young People. **SupplementaryFile 3.** Interview Guide for Support People. **Supplementary File 4.** EIPS factors that could improve thehospitalisation experience and its effectiveness – Views of Young People andSupport People.   

## Data Availability

Due to the conditions outlined in the study’s consent process, full transcripts cannot be shared to protect participant anonymity. However, amalgamated datasets generated and/or analysed during the current study are available from the corresponding author on reasonable request.

## Abbreviations

EIPS: Early intervention psychosis services
EPYS: Early psychosis youth service
FEP: First episode of psychosis
SP: Support person/people
UHR: Ultra-high risk
YP: Young person

## References

[1] Coates D, Wright L, Moore T, Pinnell S, Merillo C, Howe D (2019). The psychiatric, psychosocial and physical health profile of young people with early psychosis: data from an early psychosis intervention service. Child & Youth Services.

[2] Early Psychosis Guidelines Writing Group and EPPIC National Support Program. Australian Clinical Guidelines for Early Psychosis, 2nd edition (update). Melbourne: Orygen, The National Centre of Excellence in Youth Mental Health; 2016.

[3] EY, The University of Sydney, The George Institute (2020). Evaluation of the early psychosis Youth Services Program - Final Report.

[4] Anderson KK, Fuhrer R, Schmitz N, Malla AK (2013). Determinants of negative pathways to care and their impact on service disengagement in first-episode psychosis. Soc Psychiatry Psychiatr Epidemiol.

[5] Killackey E (2016). The EPPIC Model Integrity Tool.

[6] Bridgett M, Abrahamson G, Ho J (2015). Transition, it’s more than just an event: supporting young people with type 1 diabetes. J Pediatr Nurs.

[7] Mulvale A, Miatello A, Hackett C, Mulvale G (2016). Applying experience-based co-design with vulnerable populations: Lessons from a systematic review of methods to involve patients, families and service providers in child and youth mental health service improvement. Patient Experience Journal.

[8] Productivity Commision (2020). Mental health. Report no.95..

[9] Tonin V (2007). Young people seeking mental-health care. Lancet (London England).

[10] Thornicroft G, Tansella M (2005). Growing recognition of the importance of service user involvement in mental health service planning and evaluation. Epidemiol Psychiatric Sci.

[11] Raballo A, Poletti M, McGorry P (2017). Architecture of change: rethinking child and adolescent mental health. The Lancet Psychiatry.

[12] Singh S (2017). Early intervention in psychosis: much done, much more to do. World Psychiatry.

[13] Singh SP, Tuomainen H (2015). Transition from child to adult mental health services: needs, barriers, experiences and new models of care. World Psychiatry.

[14] Tindall RM, Simmons MB, Allott K, Hamilton BE (2018). Essential ingredients of engagement when working alongside people after their first episode of psychosis: a qualitative meta-synthesis. Early Interv Psychiat.

[15] Tindall RM, Allott K, Simmons M, Roberts W, Hamilton BE (2018). Engagement at entry to an early intervention service for first episode psychosis: an exploratory study of young people and caregivers. Psychosis.

[16] Tindall RM. Experiences of engagement with early intervention services for first episode psychosis: a longitudinal, qualitative study. Melbourne, Victoria: University of Melbourne; 2020.

[17] Tindall R, Francey S, Hamilton B (2015). Factors influencing engagement with case managers: perspectives of young people with a diagnosis of first episode psychosis. Int J Ment Health Nurs.

[18] McCann TV, Lubman DI, Clark E (2011). First-time primary caregivers’ experience accessing first‐episode psychosis services. Early Interv Psychiat.

[19] Kvale S, Brinkmann S. Interviews: Learning the craft of qualitative research interviewing. Thousand Oaks, California: Sage Publications; 2009.

[20] Archer M, Bhaskar R, Collier A, Lawson T, Norrie A. Critical realism: Essential readings. London, UK: Routledge; 2013.

[21] Buus N, Perron A (2020). The quality of quality criteria: replicating the development of the Consolidated Criteria for reporting qualitative research (COREQ). Int J Nurs Stud.

[22] Tong A, Sainsbury P, Craig J (2007). Consolidated criteria for reporting qualitative research (COREQ): a 32-item checklist for interviews and focus groups. Int J Qual Health Care.

[23] Hagaman AK, Wutich A (2017). How many interviews are enough to identify metathemes in multisited and cross-cultural research? Another perspective on Guest, Bunce, and Johnson’s (2006) landmark study. Field methods.

[24] Hennink M, Kaiser BN. Sample sizes for saturation in qualitative research: a systematic review of empirical tests. Social Science & Medicine. 2021;292:114523. 10.1016/j.socscimed.2021.114523 34785096

[25] Braun V, Clarke V (2006). Using thematic analysis in psychology. Qualitative Res Psychol.

[26] Barry CA, Britten N, Barber N, Bradley C, Stevenson F (1999). Using reflexivity to optimize teamwork in qualitative research. Qual Health Res.

[27] Drake RJ, Husain N, Marshall M, Lewis SW, Tomenson B, Chaudhry IB (2020). Effect of delaying treatment of first-episode psychosis on symptoms and social outcomes: a longitudinal analysis and modelling study. The Lancet Psychiatry.

[28] McGorry PD, Killackey E, Yung AR (2007). Early intervention in psychotic disorders: detection and treatment of the first episode and the critical early stages. Med J Aust.

[29] Hughes F, Stavely H, Simpson R, Goldstone S, Pennell K, McGorry P (2014). At the heart of an early psychosis centre: the core components of the 2014 early psychosis Prevention and intervention centre model for australian communities. Australasian Psychiatry.

[30] Cabassa LJ, Piscitelli S, Haselden M, Lee RJ, Essock SM, Dixon LB (2018). Understanding pathways to care of individuals entering a specialized early intervention service for first-episode psychosis. Psychiatric Serv.

[31] Buus N, McCloughen A. Client and family responses to an open dialogue Approach in early intervention in psychosis: a prospective qualitative case study. Issues in Mental Health Nursing. 2021;43(4):308–316. 10.1080/01612840.2021.1986758 34666592

[32] Farrelly S, Brown G, Rose D, Doherty E, Henderson RC, Birchwood M (2014). What service users with psychotic disorders want in a mental health crisis or relapse: thematic analysis of joint crisis plans. Soc Psychiatry Psychiatr Epidemiol.

[33] Law H, Morrison AP (2014). Recovery in psychosis: a Delphi study with experts by experience. Schizophr Bull.

[34] Reid R, Haggerty J, McKendry R. Defusing the confusion: concepts and measures of continuity of healthcare. Ottawa, Canada: Canadian Health Services Research Foundation, the Canadian Institute for Health Information, and the Advisory Committee on Health Services of the Federal/Provincial/Territorial Deputy Ministers of Health; 2002.

[35] Waring J, Marshall F, Bishop S (2015). Understanding the occupational and organizational boundaries to safe hospital discharge. J Health Serv Res Policy.

[36] Wright N, Rowley E, Chopra A, Gregoriou K, Waring J (2016). From admission to discharge in mental health services: a qualitative analysis of service user involvement. Health Expect.

[37] Singh SP, Paul M, Ford T, Kramer T, Weaver T, McLaren S (2010). Process, outcome and experience of transition from child to adult mental healthcare: multiperspective study. Br J Psychiatry.

[38] Leuci E, Quattrone E, Pellegrini P, Pelizza L (2020). The “Parma—Early Psychosis” program: general description and process analysis after 5 years of clinical activity. Early Interv Psychiat.

[39] Hackett CL, Mulvale G, Miatello A (2018). Co-designing for quality: creating a user‐driven tool to improve quality in youth mental health services. Health Expect.

[40] Tanskanen S, Morant N, Hinton M, Lloyd-Evans B, Crosby M, Killaspy H (2011). Service user and carer experiences of seeking help for a first episode of psychosis: a UK qualitative study. BMC Psychiatry.

