# Supplementary material for: Experiences of service transitions in Australian early intervention psychosis services: a qualitative study with young people and their supporters
Source: BMC Psychiatry. 2022 Dec 13;22:788. doi: 10.1186/s12888-022-04413-0 (PMC9749227; doi:10.1186/s12888-022-04413-0)
Supplement: Supplementary file 1 — Additional file 1: SupplementaryFile 1. COREQ checklist. SupplementaryFile 2. Interview Guide for Young People. SupplementaryFile 3. Interview Guide for Support People. Supplementary File 4. EIPS factors that could improve thehospitalisation experience and its effectiveness – Views of Young People andSupport People. [file 12888_2022_4413_MOESM1_ESM.docx]

## Supplementary Files

Supplementary File 1. COREQ checklist

Authors: 1. Alyssa Milton; 2. Tacita Powell, 3. Katrina Conn, 4. Rochelle Einboden, 5. Niels Buus, 6. Nick Glozier.

| **Number** | **Item** | **Description** | **Page Number (submitted manuscript)** |
| --- | --- | --- | --- |
| 1. | Interviewer | Author 1 and 2 conducted the interviews with young people and their support people accessing EIP services (state and federal). | 9,10 |
| 2. | Researcher credentials | Author 1: BSc, PGDip Psych, MAppSc Health Psych, PhD  Author 2: BSc, MBBS, M Psychiatry  Author 3: BSc, BEd  Author 4: BScN, MN, PhD  Author 5: BN, MScN, PhD  Author 6: MA, MBBS, MSc, MRCPsych, FRANZCP, PhD | 29 |
| 3. | Occupation | Author 1 is a psychologist and research fellow specializing in qualitative and mixed methods research and has Australian and international experience working with EIPS.  Author 2 is a psychiatry registrar (trainee) and has experience working with EIPS services.  Author 3 is a teacher and lived experience researcher  Author 4 is a nurse and qualitative researcher specializing in critical social theory.  Author 5 is a mental health nurse and specialist in qualitative research and qualitative research methodology, with a focus on critical health research, ethnographic theories and methods.  Author 6 is an academic psychiatrist/SP. | 29 |
| 4. | Gender | Authors 1, 2, 3 and 4 are female. Authors 5 and 6 are male. | 7 |
| 5. | Experience and training | Authors are experienced and active researchers with expertise in qualitative, quantitative, and mixed methods approaches. Authors have researched and published in the broad topic area previously. | 6,7,9,11,29 |
| 6. | Relationship established | A two-stage consent process was applied, where clinicians briefly described the study to potential participants meeting eligibility criteria gaining consent to refer to the researcher. The clinician scheduled the interview for those expressing interest in participating. Prior to interview, participants had the opportunity to review the participant information and consent forms, and discuss any questions, before giving informed consent. Participants aged 12-18 years required parental/guardian co-consent, with 16-18 year olds parental/guardian consent being subject to clinician advice and state specific laws. | 9 |
| 7. | Participants’ knowledge of the interviewer | All interviewers had no previous professional or personal relationship with any of the participants. Co-authors only reviewed de-identified data thus had no personal relationship with, or knowledge of participants. Participants were informed about who the involved researchers were in the Participant Information Statement (PIS). | 10 |
| 8. | Interviewer characteristics | The interviewers were conducted by a psychologist and researcher with experience in qualitative and mixed-methods research in Australian and international mental health services including EIPS (Author 1) and a psychiatry registrar with experience in community based EIPS, pediatric and adult mental health services and hospital services (author 2). Interviews were supported by the research team which included Author 3 who is a researcher with lived experience of using EIPS as a client. | 9,10 |
| 9. | Methodological orientation and theory | Thematic analysis with an inductive approach was applied, using a critical realist orientation. | 6 |
| 10. | Sampling | To enable participation in the research, clinicians were asked to nominate and invite all clients (and their SPs) on their caseload who met eligibility criteria and had capacity to consent. Purposive sampling was used to recruit a diverse sample of the EIPS client population and their support people (family, partners or carers) who had an experience with the hospital system either personally or via the young person they support. Representation from special interest groups was prioritised. Special interest group of clients and family / carers represented various clinical stages (UHR, FEP), ages, genders, culturally and linguistically diverse and Aboriginal or Torres Strait Islander backgrounds. Sampling did not require researcher access to client records. The researchers fed back to the coordinating clinicians at each EIPs if there were any recruitment gaps and clinicians checked their entire caseload for eligibility so as to minimise the potential for gatekeeping and bias. | 8,9 |
| 11. | Method of approach | Eligible participants were recruited through clinician referral. EIPS clinicians and managers, spoke of the study to potential participants, sent communications to clients and support people advising of the study. Clinicians invited all eligible young people and support people on their caseload who met criteria and had capacity to consent. | 8 |
| 12. | Sample size | Semi-structured interviews were conducted with 27 YP and 12 SP accessing EIPS. | 11 |
| 13. | Non-participation | One young person chose not partake in the interview after reading the participant information statement. All interviews that commenced were completed. The number of participants who declined at clinician invitation was not recorded. | 11 |
| 14. | Setting of data collection | Interviews were conducted face-to-face on the EIPS premises or via telephone between Dec 2019 and May 2020. | 9 |
| 15. | Presence of non-participants | Participants could choose to have a support person or advocate present at the interview (who could also consent and contribute to the interview), however this was not required for any interviews. Two YP requested a support person to be present who also participated in the interviews. | 9, 11 |
| 16. | Description of sample | A diverse sample of young people and their support people (parents or partners) receiving support from Australian state or federally funded EIPS.  Participants were YP and their SP accessing a participating EIPS. The eligibility criteria for young people included: (1) aged 12-25 years; (2) clinician nominated; (3) minimum two week service engagement; (4) provided parent or guardian if aged between 12-15 years, and when advised by the clinician, aged between 16-18 years. The eligibility criteria for family or carers included: (1) being 18 years of age or over; and (2) being a parent, guardian, family member or friend of a current EIPS client. | 7, 8, 11, Table 1 |
| 17. | Interview guide | Interviews were semi-structured. Questions covered: Client experience of coming into the program; Client experience of the program; Client views on the impact of the program on their functional outcomes; Client hospitalisation experience whilst involved in the program; Treatment (medication, CBT, family care); Ongoing community care, mobile outreach and group programs; Family programs and family peer support; Youth participation and peer support program. In order to enhance question relevance, a lived experience researcher who had used EIPS contributed to the interview design. | 7,9, Supplementary File 2 & 3 |
| 18. | Repeat interviews | NA | NA |
| 19. | Audio/visual recording | Interviews were audio-recorded. | 9 |
| 20. | Field notes | NA | NA |
| 21. | Duration | The average interview duration was 57 minutes for YP and 68 minutes for SPs. | 9,10 |
| 22. | Data saturation | Ensuring sufficient numbers of participants were recruited for the purposes of theme saturation was guided by Hagaman and Wutich (2017) and Hennick et al. (2017) indicating 20-40 participants would be required. This saturation guidance was selected as the research involved recruiting a non-homogenous participants (ie. both YP and SP) and was run across multiple EIPS settings across Australia with different funding (state and federal). As it was part of a larger evaluation, we were not able to assess data saturation via a stopping criterion, however, we as described above relied on a priori estimates of an appropriate sample size to reach saturation. | 9,10 |
| 23. | Transcripts returned | Transcripts were not returned to participants. A lay-summary of findings was returned to participants. | 11 |
| 24. | Number of data coders | Data was coded by authors 1 and 3 and supported by MY (acknowledgements). | 10,11 |
| 25. | Description of the coding tree | Codes, themes, and subthemes were iteratively refined and developed during regular research team meetings between with authors 1, 2 and 3 (MY and AC in the acknowledgments) and were triangulated with the other authors. Descriptions of the themes, subthemes and codes were developed and captured in a coding framework and codebook facilitated in Nvivo 12 software. | 10,11 |
| 26. | Derivation of themes | Themes and subthemes were derived from the data using iterative inductive processes. | 10,11 |
| 27. | Software | NVivo 12 | 10 |
| 28. | Participant checking | Participant checking did not take place. Instead the lived experience researcher were involved in the coding and theme identification process to enhance validity of the interpretation. Further, outside of the lay summary of the findings being returned, there was no formal opportunity for participants to feedback on the findings and recommendations other than contacting the researchers directly. | 11 |
| 29. | Quotations presented | Illustrative quotes from participants were used. Quotations are identified with participant type and number to ensure anonymity. Specifically, YP = young person and SP = support person. | 11-20, Supplementary File 4 |
| 30. | Data and findings consistent | Data and findings are consistent throughout the manuscript. Young People and Support People provided perspectives on EIPS support and there were many thematic similarities in their data. Accordingly, findings are presented together. Differences in participants’ (YP and SP) experiences accessing state and federally funded EIPS were highlighted where appropriate. | 11-20 |
| 31. | Clarity of major themes | There were four major service related transition points in a young person’s journey with the EIPS identified that reflected critical moments of care and its continuity. These included 1) *transitioning into EIPS;* 2) *within service changes; 3) transitioning in and out of hospital whilst in EIPS care; and, 4) EIPS discharge.* Transition themes are clearly described and presented via main headings in the results section and visualized in Figure 1. | 11-20 |
| 32. | Clarity of minor themes | There were four EIPS program delivery sub-themes that spanned across all service transition points. These included *1) continuity of care; 2) service accessibility and flexibility; 3) person centredness; and, 4) bio-psychosocial support.* These are presented in Figure 1 as sub themes, are discussed throughout the results section and contextualized in relation to each transition point theme in the discussion section. | Figure 1, 11-20 |

Supplementary File 2. Interview Guide for Young People

| **Concept** | **Topic** | **Possible Questions** |
| --- | --- | --- |
| Pathway to care  (access and expectations) | 1. Client experience of coming into the program | 1. *Can you please tell me about what led up to you becoming involved with this program?*  - *Researcher to confirm whether referral to service was via hospital. If yes, what was that experience like for you? If not, can you tell me about the process of coming into the program? Prompt: How long did it take between having some problems and then engaging with hYEPP (headspace Youth Early Psychosis Program)/EIPS?*  1. *What were you hoping to get out of the program? Prompt: What did you think you needed from the program?* |
| Engagement and perception of programs appropriateness | 1. Client experience of the program | 1. *Can you please tell me about the types of problems or issues that the program has helped you to manage?*  - *Prompt: What types of support or care have you received from the program? Are you able to access care when you need it?*  1. *What aspects of the program work well or not so well for you? Prompt: Are you able to access care/support when you need it?* |
| Functional Outcomes associated with the program | 1. Client views on the impact of the program on their functional outcomes | 1. *We are interested in hearing your thoughts on the impact of the hYEPP/EIPS program on different areas of your life. Can you think of anything that has changed for you since accessing the program?*  - *Prompt if requiring further examples: This might include areas of your life such as employment or education, your relationships or social life, your leisure activities, your daily living skills (like looking after yourself, managing your finances, looking after your home)*  1. *For each outcome mentioned ask: Can you tell me what this change means for you personally?*  - *Prompt: is it important or not? Why is that?*  1. *Given these changes, can you tell me how you continue to manage your mental health or any symptoms? Probe: self-management, personal understanding of situation/ diagnosis, support networks.* 2. *How was the hYEPP/EIPS staff/service involved in these changes in your life?* 3. *Were any other services or people involved in these changes?* |
| Hospitalisation experiences | 1. Client hospitalisation experience whilst involved in the program | 1. *Did you have any hospital admissions while receiving hYEPP/EIPS care?*  - *Prompt: If yes explore… how many times were you in hospital? Was it a voluntary or an involuntary admission? What was the impact of this experience on your engagement with services?*  1. *What was the process of going into hospital, being in hospital and transitioning out of hospital like for you?* 2. *What was the support from the hYEPP/EIPS staff like for you during this time?*  - *Prompt: Can you tell me how you found the communication between you, the hospital staff and the hYEPP/EIPS service?* - *Prompt: Can you tell me how you felt about your involvement in your own care?* - *Prompt: What aspects of your care did you focus on with the hYEPP/EIPS service at this time?*  1. *Overall, do you feel that hYEPP/EIPS involvement was helpful or not helpful to you during this time? Why is that?*  - *Prompt: Is there anything the hYEPP/EIPS team did particularly poorly or particularly well?* - *Prompt: Can you think of anything else you would have liked from the hYEPP/EIPS team at this time?*  1. *Have you had any hospitalisation experiences when you were not with the hYEPP/EIPS program? If yes, how do these experiences compare?* 2. *Could you tell us whether you feel that being a part of the hYEPP/EIPS program impacted the length of your hospital stay or your admission to hospital in anyway?* |
| *Specific Fidelity Topics*  *Note: these topics may already have been addressed by participants in response to Question B. If not, these topics may be discussed.* | | |
| Fidelity | 1. Treatment (medication, CBT, family care) | 1. *Can you please tell me about any treatments you were offered or received? (What, When, How, Who) Prompt: How are decisions about your treatment made?* 2. *What aspects of treatment worked well or not so well for you?* |
|  | 1. Ongoing community care, mobile outreach and group programs | 1. *Can you tell me about the kinds of service and care you have received? (Who, What, When, Why) Prompts: Have you ever needed crisis support or care after hours? Have you attended any group programs?* 2. *What aspects of these types of services work well or not so well for you?* |
|  | 1. Family programs and family peer support | 1. *Have any of your family, carers or friends participated in any of the family programs or family peer support programs? (What, When, How, Who)* 2. *Can you tell me how their involvement in the program has been helpful to you, or not?* |
|  | 1. Youth participation and peer support program | 1. *Can you please tell me about any involvement you had with the youth participation and peer support programs? (What, When, How, Who)* 2. *Do they sound interesting or appealing to you?* 3. *What aspects of these programs work well or not so well for you?* |

Supplementary File 3. Interview Guide for Support People

| **Concept** | **Topic** | **Possible Questions** |
| --- | --- | --- |
| *Core Topics* | | |
| Pathway to care (access and expectations) | 1. Family/carer experience of coming into the program | 1. *Can you please share how you found out about this program?* 2. *Can you please share your story about coming into the program?*  - *Researcher to confirm whether referral to service for young person was via hospital. If yes, what was that experience like for you/ the young person you were supporting? If not, can you tell me about the process of coming into the program? Prompt: How long did it take between first noticing the young person you care for having trouble and connecting with hYEPP/EIP?*  1. *What were you hoping to get out of the program? Prompt: What did you think you and/or the young person you care for needed from the program?* |
| Engagement and perceptions of program appropriateness | 1. Family/carer experience of the program | 1. *Can you please share a few examples of the types of problems or issues that the program has helped you manage? Prompt: What types of support have your received from the program?* 2. *What aspects of the support provided work well or not so well for you? Prompt: Are you able to access support when you need it?* |
| Functional Outcomes associated with the program | 1. Client views on the impact of the program on their functional outcomes | 1. *We are interested in hearing your thoughts on the impact of the hYEPP/EIP program on different areas the young person you support’s life. Can you think of anything that has changed for them since accessing the program?*  - *Prompt if requiring further examples: This might include areas of their life such as employment or education, their relationships or social life, their leisure activities, their daily living skills (like looking after themselves, managing finances, looking after their home)*  1. *For each outcome mentioned ask: Can you tell me what this change means for you/ them?*  - *Prompt: is it important or not? Why is that?*  1. *Given these changes, can you tell me how the young person you support continues to manage their mental health or any symptoms? Probe: self-management, personal understanding of situation/ diagnosis, support networks.* 2. *How was the hYEPP/EIP staff/service involved in these changes in their life?* 3. *Were any other services or people involved in these changes?* |
| Hospitalisation experiences | 1. Family/carer hospitalisation experience whilst involved in the program | 1. *Did the young person you support have any hospital admissions while receiving hYEPP/EIP care?*  - *Prompt: If yes explore… how many times were they in hospital? Was it a voluntary or an involuntary admission? What was the impact of this experience on your/ their engagement with services?*  1. *What was the process of the young person you support going into hospital, being in hospital and transitioning out of hospital like for you?* 2. *What was the support from the hYEPP/EIP staff like for you/ them during this time?*  - *Prompt: Can you tell me how you found the communication between you, the hospital staff and the hYEPP/EIP service?* - *Prompt: Can you tell me how you felt about your involvement in their care?* - *Prompt: What aspects of the young person you support’s care did you focus on with the hYEPP/EIP service at this time?*  1. *Overall, do you feel that hYEPP/EIP involvement was helpful or not helpful to you/ the young person you support during this time? Why is that?*  - *Prompt: Is there anything the hYEPP/EIP team did particularly poorly or particularly well?* - *Prompt: Can you think of anything else you would have liked from the hYEPP/EIP team at this time?*  1. *Did the young person you support have any hospitalisation experiences when you were not with the hYEPP/EIP program? If yes, how do these experiences compare?* 2. *Could you tell us whether you feel that your family being a part of the hYEPP/EIP program impacted the length of the young person you support’s hospital stay or their admission to hospital in anyway?* |
| *Specific Fidelity Topics*  *Note: these topics may already have been addressed by participants in response to Question B. If not, these questions may be asked.* | | |
| Fidelity | 1. Treatment (medication, CBT, family care) | 1. *Can you please share what treatments the young person you care for was offered or received? (What, When, How, Who) Prompt: How are treatment decisions made?* 2. *What aspects work well or not so well for you and the young person you care for?* |
|  | 1. Ongoing community care, mobile outreach and group programs | 1. *Can you please share the kinds of support you have received? (Who, What, When, Why) Prompts: Has the young person you care needed crisis support or care after hours? Has the young person you care for attended group programs?* 2. *What aspects of this community support work well or not so well for you and the young person you care for?* |
|  | 1. Family programs and family peer support | 1. *Have you or any other family members, carer or friends participated in any of the family programs or family peer support programs? (Who, What, When, Why)* 2. *Can you share how these programs were helpful to you or the young person you care for, or not?* |
|  | 1. Youth participation and peer support program | 1. *Has the young person you care for visited the youth participation and peer support programs? (Who, What, When, Why)* 2. *What aspects of these programs work well or not so well for you and the young person you care for?* |

**Supplementary File 4.** EIPS factors that could improve the hospitalisation experience and its effectiveness – Views of Young People and Support People

|  |  | **Illustrative quotes** | |
| --- | --- | --- | --- |
| *Context* | *Participant* | *Higher level integration* | *Lower level integration* |
| Geographical | SP P1 | *Quite quickly, my son was in the hospital. They already, someone is there regularly, the MAT team is in there regularly. I'm not sure whether they already, because in the area, headspace is in the area. I'm not sure how they were in this system, but they're actually in the hospital. And I don't need to go for look for, they already offer. They got some kind of like headspace for the young people. If they're adult they've got community mental health team. They do approach to us in the hospital.* | *My daughter is in the episode of bipolar and my son is different, other illness, in, because my son was went into the [nearby] hospital which is, he was in the area. Yeah. But my daughter was the first to went into [hospital on other side of Sydney] but headspace wasn’t there (…)*  *I don't know what kind of service I can got because there is, hospital on other side of Sydney] is out of my area (…) I don't know the kind of service I can get. Like, what is if she is discharged from the hospital, what can I do? But since I know headspace, So I immediately, they said, oh, if your daughter is stable and she can discharge, if it's, send her back to, send her back to your area.* |
| Organizational | SP P3 | *We use to have a weekly meeting with the team, I guess at [hospital 1] so the psychiatrist and the nurse and what have you. And there was pretty much always a representative from Headspace there. Every Monday, I thought it was it was good to have a team of people because this was all very new to me (…) And it was good to know that when we got out, we weren't just being left. Yeah. And it was good to know that the people we were being left to had that history.* | *So they [hospital 2] didn't even know about Headspace. Pretty much headspace turned up and called them. So if that was [my son]'s first visit and we had been to another facility, I wouldn't have known about Headspace.* |
| Temporal | YP P12 | ***Participant:*** *They [headspace] came in few times to check on me… A couple times a day. Well, not couple times a day, a couple times a week (…) I found it very calming. Knowing I could share my whole story. Like I consider a whole information about myself by piece by piece.*  *(…)* ***Interviewer:*** *And so you were in hospital for a fair amount of time? A little bit of time?*  ***Participant:*** *Six months.* | ***Participant:*** *A couple of times I’ve been in hospital. But sometimes it’s been more successful not staying in hospital (…) Short, like nights or days stay in hospital (…) Their isolation room was destroyed. Very poor staff members. The staff there, being treated disappointingly, and my stuff. Yeah, they’ll get there. Sometimes we clients come in from mental wards and just want to go home. [****… Interviewer:*** *Right. And So going in, two very different experiences. But how was Headspace involved in those times? Did they come and visit?]* *They checked on me after I was in hospital.* ***[Interviewer:*** *Okay, So you've already been discharged by the time that headspace checked on you?]*  *Yeah. See how I am seeing what happened and tell me how I can do better next time. [****Interviewer:*** *Would you like to have contact with them whilst you were in hospital?]* *No because one, there's no privacy you get from hospitals. Literally none. I didn't like [hospital] at all (…) [hospital] are like poorly mistreated, unwell.* ***[Interviewer:*** *Right, do you think if headspace had spoken to you at all, they could've helped that situation? Or was it...]* ***Participant:*** *Yes, Maybe moved me to a different hospital.* |
| Individual | YP P11 | *[during the first hospital admission] I feel like the hospital staff and headspace staff knew each other well and that they could understand that the medication needs to be changed or that she has to understand the techniques better. So that was good (…) At the time my case manager changed, Sorry, at the time my case manager changed. So I had to re-explain everything. But the case manager that I had before that I had at the time was really, really good for me.* | *[in a subsequent hospital admission]* *and so then they took me to the hospital and then I had to explain the whole situation about the HSC to a psychologist. So I felt like it was needed that headspace was supposed to tell the hospital what was the scenario. But because Headspace didn't tell the psychologist, the inpatient psychologist about what happened. I had to explain the whole situation again. Which was really tiresome.* |
